# iProPhos: A Web-Based Interactive Platform for Integrated Proteome and Phosphoproteome Analysis

**DOI:** 10.1016/j.mcpro.2023.100693

**Published:** 2023-12-12

**Authors:** Jing Zou, Ziran Qin, Ran Li, Xiaohua Yan, Huizhe Huang, Bing Yang, Fangfang Zhou, Long Zhang

**Affiliations:** 1 The Second Affiliated Hospital and Life Sciences Institute and School of Medicine, The MOE Key Laboratory of Biosystems Homeostasis and Protection and Zhejiang Provincial Key Laboratory for Cancer Molecular Cell Biology, Zhejiang University, Hangzhou, China; 2 School of Medicine, Hangzhou City University, Hangzhou, China; 3 Department of Biochemistry and Molecular Biology, School of Basic Medical Sciences, Nanchang University, Nanchang, China; 4 The Second Affiliated Hospital of Chongqing Medical University, Chongqing, China; 5 Department of Pharmaceutical Chemistry and the Cardiovascular Research Institute, University of California, San Francisco, California, USA; 6 Institutes of Biology and Medical Sciences, Soochow University, Suzhou, China; 7 Cancer Center, Zhejiang University, Hangzhou, China

**Keywords:** proteomics, phosphoproteomics, integrated analysis, web server, tumor

## Abstract

Large-scale omics studies have generated a wealth of mass spectrometry–based proteomics data, which provide additional insights into disease biology spanning genomic boundaries. However, there is a notable lack of web-based analysis and visualization tools that facilitate the reutilization of these data. Given this challenge, we present iProPhos, a user-friendly web server to deliver interactive and customizable functionalities. iProPhos incorporates a large number of samples, including 1444 tumor samples and 746 normal samples across 12 cancer types, sourced from the Clinical Proteomic Tumor Analysis Consortium. Additionally, users can also upload their own proteomics/phosphoproteomics data for analysis and visualization. In iProPhos, users can perform profiling plotting and differential expression, patient survival, clinical feature–related, and correlation analyses, including protein–protein, mRNA-protein, and kinase-substrate correlations. Furthermore, functional enrichment, protein–protein interaction network, and kinase-substrate enrichment analyses are accessible. iProPhos displays the analytical results in interactive figures and tables with various selectable parameters. It is freely accessible at http://longlab-zju.cn/iProPhos without login requirement. We present two case studies to demonstrate that iProPhos can identify potential drug targets and upstream kinases contributing to site-specific phosphorylation. Ultimately, iProPhos allows end-users to leverage the value of big data in cancer proteomics more effectively and accelerates the discovery of novel therapeutic targets.

## Highlights

• iProPhos performs integrated proteome and phosphoproteome analysis.
• iProPhos provides optional parameters for plotting and statistical analysis.
• iProPhos allows users to upload their own data for analysis and visualization.
• iProPhos can identify potential drug targets and crucial phosphorylation events.

## Graphical Abstract

**Figure:**
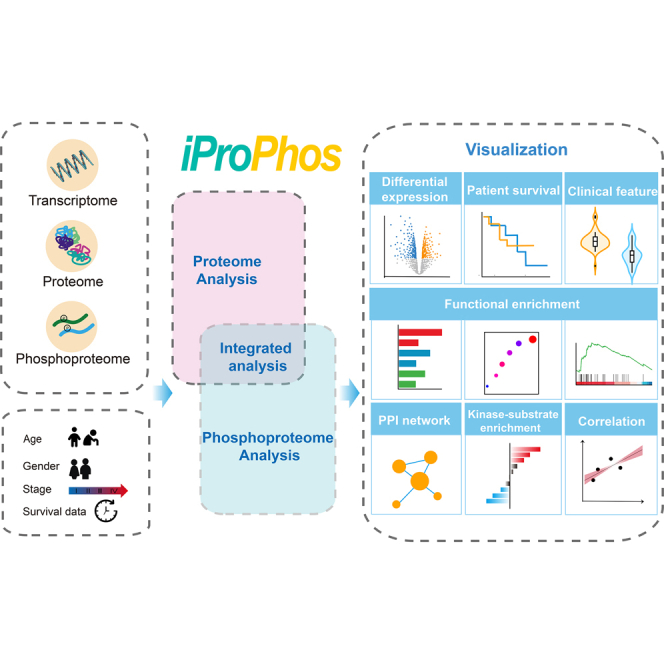

## In Brief

We have developed iProPhos, a user-friendly web server for integrated proteome and phosphoproteome analysis using CPTAC data for 12 cancer types. iProPhos has the capability of hosting abundant analyses such as differential expression, patient survival, clinical feature–related, kinase-substrate enrichment, and correlation analyses, including protein–protein, mRNA-protein, and kinase-substrate correlations. In addition, iProPhos provides functional enrichment and PPI network analyses. We present two case studies to demonstrate that iProPhos can identify potential drug targets and upstream kinases contributing to site-specific phosphorylation.

Proteins execute various biological processes and carry biological information not accessible through genomics or transcriptomics (1, 2, 3). Protein phosphorylation is one of the most common and essential posttranslational modifications (PTMs) in biological systems, considerably affecting protein function, stability, and interactions with other cellular components (4, 5, 6). Weak or negative correlations between mRNA and protein abundance in certain genes hinder gene function predictions using transcriptomic data (7, 8). With advancements in high-throughput technologies, MS-based proteomics has become a powerful tool in functional research (9, 10, 11). Large consortium projects, such as the Clinical Proteomic Tumor Analysis Consortium (CPTAC), have generated a tremendous amount of proteomic data. However, analyzing and mining these data require proficient programming skills, making proteomics analysis and visualization a considerable challenge for experimental and clinical personnel.

To date, several tools have been developed for proteomics analysis, including TCPA (12), CPPA (13), UALCAN (14) and LinkedOmics (15), but they offer a limited range of analytical types. TCPA is a web-based resource for accessing, analyzing, and visualizing cancer functional proteomics. However, it only encompasses approximately 300 reverse phase protein array–based protein markers and lacks normal samples for differential expression analysis. CPPA is an interactive web server for mining the characteristics of proteins and protein phosphorylation, but it cannot perform integrated analysis between proteomics and phosphoproteomics data. UALCAN and LinkedOmics are designed for the integrative analysis of multiomics data. However, UALCAN can only conduct differential expression analysis for proteomics data, while LinkedOmics focuses on exploring associations between different types of attributes, and lacks the capability to identify differentially expressed proteins in tumors and perform functional enrichment analysis for them. Furthermore, TCPA, CPPA, and UALCAN do not support functional enrichment analysis and cannot evaluate the correlation between mRNA and protein abundance. None of these tools provide kinase-substrate correlation analysis or protein–protein interaction (PPI) network analysis. More importantly, none of them allow users to upload their own proteomics/phosphoproteomics data for analysis, and customized options for analyzing and visualizing data are limited.

**Fig. 1:**
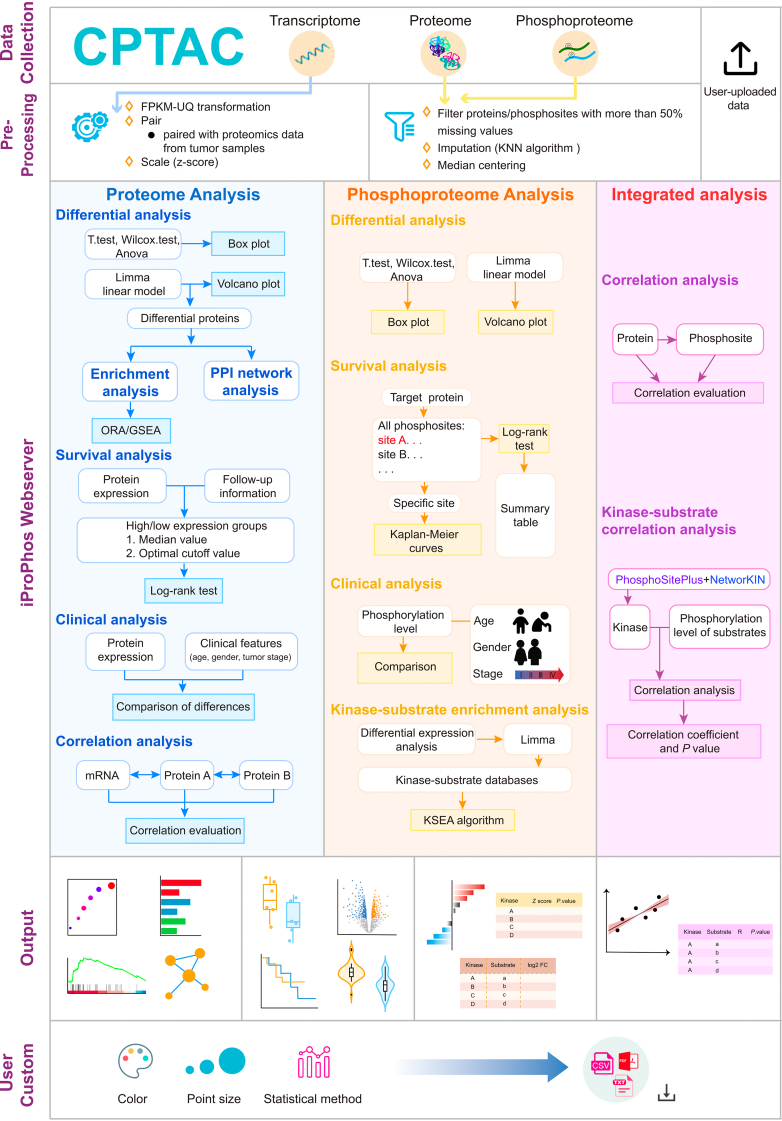
**Schematic of iProPhos.** iProPhos collected the omics data from the CPTAC project. All datasets were uniformly processed with a standardized workflow. iProPhos also allows users to upload their preprocessed data for analysis. iProPhos has analysis modules including “Proteome Analysis” and “Phosphoproteome Analysis,” as well as their integrated analysis. The *bottom part* shows the representative outputs of each module. iProPhos allows users to customize graphic parameters and statistical methods. CPTAC, Clinical Proteomic Tumor Analysis Consortium; FC, fold change; GSEA, gene set enrichment analysis; ORA, over-representation analysis; PPI, protein–protein interaction.

To address the issues with the current proteomics data analysis tools, we have developed iProPhos, a user-friendly web server for integrated proteome and phosphoproteome analysis using CPTAC data for 12 cancer types, comprising 1444 tumor samples and 746 normal samples (supplemental Table S1). Furthermore, iProPhos allows users to upload their preprocessed proteomics/phosphoproteomics data for analysis and visualization. iProPhos has “Proteome Analysis” and “Phosphoproteome Analysis” modules (Fig. 1). These modules perform similar functions for differential analysis, survival analysis and clinical feature-related analyses (including age, gender, and tumor stage). In the “Proteome Analysis” module, iProPhos performs functional enrichment analysis on proteins of interest and incorporates transcriptomics data paired with proteomics data for each sample to conduct correlation analysis between mRNA and protein abundance. In the “Phosphoproteome Analysis” module, iProPhos can infer kinase activity changes in tumors and integrate proteomics and phosphoproteomics data for correlation analysis. Moreover, iProPhos allows users to customize the analytic parameters, graphic color, and point size. Table 1 compares the key features of iProPhos with those of the other available tools. Overall, iProPhos overcomes the limitations of existing proteomics analysis tools by integrating proteomics with transcriptomics or phosphoproteomics, providing downstream functional enrichment analysis and PPI network analysis, as well as customized data visualization options. These distinguishing features considerably broaden the scope of applications of iProPhos.

**Table 1:** Comparison of iProPhos with existing web servers

Feature	TCPA	CPPA	UALCN	LinkedOmics	iProPhos
Data type	RPPA- based	MS-based	MS-based	RPPA- and MS-based	MS-based
Differential expression analysis	-	√	√	-	√
Functional enrichment analysis	-	-	-	-	√
PPI network analysis	-	-	-	-	√
mRNA-protein correlation	-	-	-	√	√
Survival analysis	√	√	-	√	√
Kinase-substrate correlation	-	-	-	-	√
Clinical features–related analysis	√	√	-	√	√
User upload	-	-	-	-	√
Customized options	-	-	-	-	√

Symbols used for feature evaluations with “√” for present, “-”for absent. The URLs for each tool are given below.

TCPA: https://www.tcpaportal.org/tcpa/.

CPPA: http://cppa.site/cppa/.

UALCAN: https://ualcan.path.uab.edu/.

LinkedOmics: http://linkedomics.org/.

iProPhos: http://longlab-zju.cn/iProPhos/.

Abbreviations: MS, mass spectrometry; RPPA, reverse phase protein array.

To test iProPhos and demonstrate its capabilities, we presented two case studies and showed that iProPhos had good performance in investigating potential drug targets and revealing crucial phosphosites and their upstream kinases. In summary, iProPhos can provide new insights into the mechanisms underlying cancer progression and facilitate the exploration of novel therapeutic strategies for researchers.

## Experimental Procedures

### Data Collection

Transcriptome, proteome, phosphoproteome, and clinical characteristic data of 12 cancer types were sourced from the CPTAC data portal (https://proteomic.datacommons.cancer.gov/pdc/) (16). The expression levels of the transcripts were quantified using the normalized metric, FPKM-UQ (https://docs.gdc.cancer.gov/Data/Bioinformatics_Pipelines/Expression_mRNA_Pipeline/). Protein abundance and phosphorylation levels were uniformly processed using the common data analysis pipeline (17). Clinical characteristics included age, gender, tumor stage, survival status, and survival time. In addition, we collected proteomics and phosphoproteomics data from matched normal samples, as well as transcriptomics data paired with proteomics data from tumor samples.

### Data Preprocessing

For the transcriptomics data, we centered and scaled the normalized gene expression data to generate standardized expression values (z-scores). In the proteomics data, we filtered proteins with more than 50% missing values across samples and imputed missing values using KNN algorithm implemented in the R package impute. The principles of the filter and imputation method for missing values were also applied to the phosphoproteomics data. Proteomics and phosphoproteomics data were tandem mass tag–based, and the relative abundances of proteins or phosphosites are presented as log2 tandem mass tag ratios with the median centering.

### Implementation

The iProPhos web server is freely available to all users without login requirement for access. We built it on the R Shiny framework, using the HTML5, CSS, and Shiny JavaScript (shinyjs) libraries for rendering and interactive operations of front-end pages. The user interface layout is organized with a sidebar containing widgets for target inputs and options for analysis and visualization. The main panel displays the outputs in plot or table formats. Interactive tables were generated using the R package DT (https://cran.r-project.org/web/packages/DT/index.html) to facilitate efficient data querying and selection. All outputs are available for download, with plots saved in PDF format as editable vector graphics compatible with vector graphic editors, such as Adobe Illustrator. iProPhos has been successfully tested on several browsers, including Google Chrome, Firefox, Internet Explorer (version 10 or later), and Safari. The R packages used in the web server are listed in supplemental Table S2.

## Results

### iProPhos Performs Proteomics-Related Analyses

iProPhos can perform proteomics-related analyses and integrates transcriptomics data to investigate the complex relationships between gene expression and protein abundance in biological systems.

**Fig. 2:**
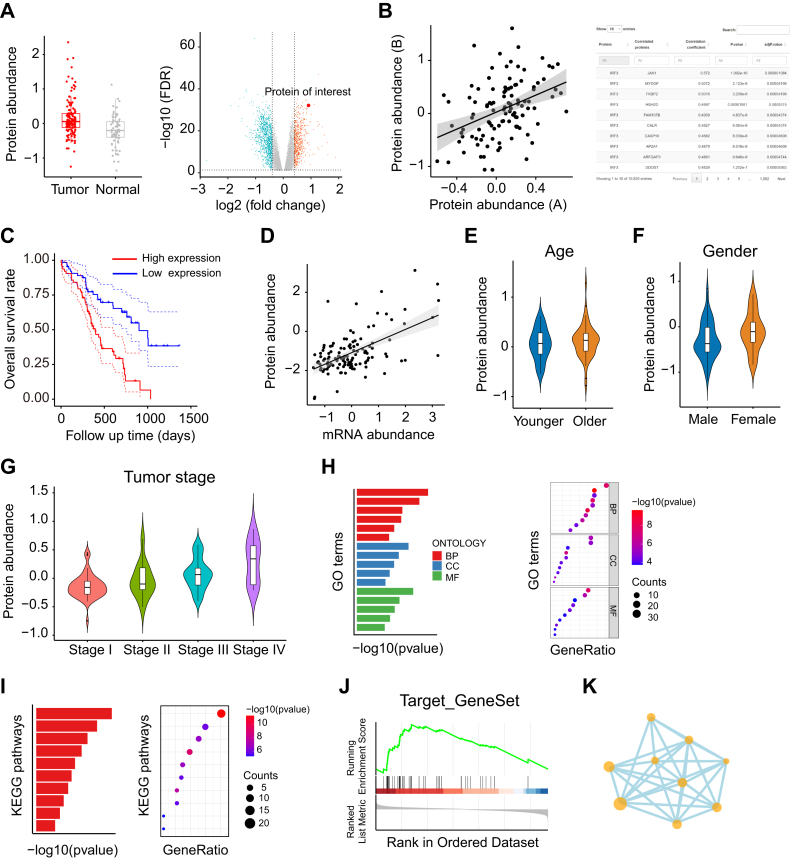
**Proteome analysis module of iProPhos.***A*, iProPhos presents differential expression analysis results with *boxplots* or *volcano plots*. *B*, the *scatter plot* illustrates the correlation between the abundance of two interested proteins, and the table displays the correlation of the abundance of one interested protein with the other detectable proteins in a given tumor type. *C*, Kaplan–Meier curves for overall survival. Patients are grouped based on the abundance of the protein of interest. *D*, iProPhos provides correlation analysis between mRNA and protein abundance. *E*–*G*, iProPhos provides clinical feature–related analysis for protein abundance, including age (*E*), gender (*F*), and tumor stage (*G*). *H*, iProPhos presents gene ontology (GO) enrichment analysis results with *bar plots* or *dot plots*. *I*, iProPhos presents Kyoto Encyclopaedia of Genes and Genomes (KEGG) pathway enrichment analysis results with *bar plots* or *dot plots*. *J*, iProPhos can perform GSEA analysis for the target gene set. *K*, the PPI network map of proteins of interest. BP, biological process; CC, cell component; GSEA, gene set enrichment analysis; MF, molecular function; PPI, protein–protein interaction.

Differentially expressed proteins between tumor and normal samples drive tumorigenesis and progression. iProPhos conducts differential expression analysis using two approaches and presents the results with box plots and volcano plots (Fig. 2*A*). For the box plots, users can choose *t* test, Wilcoxon test, or ANOVA methods for hypothesis testing to evaluate the differences between two groups of samples. The *t* test is two-tailed and assumes unequal variances, and the Wilcoxon test is a Wilcoxon rank-sum test. The box color and point size are customizable. For the volcano plots, iProPhos fits a linear model to calculate the fold change and statistical significance of proteins using the R package limma (18). Users can also set the cutoff value to define significance. Upregulated and downregulated proteins in tumor samples are labeled orange and blue respectively, whereas gray indicates nonsignificance. Moreover, a protein of interest can be magnified and highlighted with its gene symbol. The interactive table is displayed under a volcano plot containing the fold change and statistical significance data.

Correlation analysis is essential for evaluating cooperative relationships between any two proteins. iProPhos delivers correlation results in the form of scatter plots or tables (Fig. 2*B*), allowing users to explore protein correlations conveniently. In the “Correlation Plot” tab panel, users can investigate correlations between two proteins of interest in a specific tumor and customize various graphic parameters (such as point size and color) and correlation measuring methods (*e.g.*, Pearson, Spearman, and Kendall correlations). In the “Correlation Table” tab panel, users can investigate correlations of the target protein with other proteins to gain biological ideas for further study.

Survival analysis can associate protein abundance with clinical outcomes and provide valuable insights for selecting potential drug targets. iProPhos provides two methods for calculating the cut-off value that divides patients into two groups. One method is to set the median value of protein abundance as the cutoff point, and the other is to utilize the surv_cutpoint algorithm applied in the R package survminer to determine the optimal cut-off point. The colors for these two groups are also customizable. iProPhos outputs Kaplan–Meier survival curves with log-rank *p* values and 95% confidence intervals (Fig. 2*C*).

Proteins are functionally relevant to phenotypes. However, the extent to which protein abundance correlates with the mRNA level and whether this correlation breaks down in some genes are not fully understood. mRNA-protein correlation analysis can refine our understanding of the principles of gene expression control in tumor cells (19). iProPhos can perform correlation analysis between given mRNA and protein in the specific cancer type (Fig. 2*D*). The point size, color, and analysis methods are customizable.

iProPhos can investigate associations between protein abundance and clinical features, such as age, gender, and tumor stage (Fig. 2, *E*–*G*), which are crucial for identifying potential prognostic biomarkers and advancing personalized medicine. For age-relevant analysis, iProPhos allows users to choose the patients’ median age or to input a suitable number as the cutoff value. These analyses output the results with violin plots. The methods for differential analysis are optional, except for tumor stage-related analysis, where one-way ANOVA is required.

Enrichment analysis focuses on gene sets that share common biological attributes, helping researchers identify dysregulated pathways and generate hypothesises (20, 21). iProPhos performs over-representation analysis and gene set enrichment analysis (GSEA) using the R package clusterProfiler (22). For over-representation analysis analysis, users can set a cut-off value to define differentially upregulated or downregulated proteins. Then functional enrichment analysis is performed based on Gene Ontology (23, 24) or Kyoto Encyclopedia of Genes and Genomes (25, 26) annotations. Redundant Gene Ontology terms are removed using “simplify” method to present the most informative terms. The results are output in the table format and displayed with bar plots and dot plots (Fig. 2, H and I). For GSEA analysis, users can input the gene list of interest and rank genes based on the fold change from the differential expression analysis. The GSEA result is shown graphically, along with statistical significance (Fig. 2*J*).

PPI networks represent the relationships between proteins that are crucial for deciphering the mechanisms of cellular processes and diseases. iProPhos performs PPI network analysis using the R package stringdb (27). Users can customize the cut-off value to define proteins for network analysis. iProPhos also generates an interactive network diagram of protein interactions (including both physical and functional interactions) (Fig. 2*K*). Nodes represent proteins, and edges represent their interactions, with thicker edges indicating a higher confidence of interactions. Additionally, users can download the results in tabular format for further analysis in Cytoscape (https://cytoscape.org/) or other similar software.

### iProPhos Performs Phosphoproteomics-Related Analyses

**Fig. 3:**
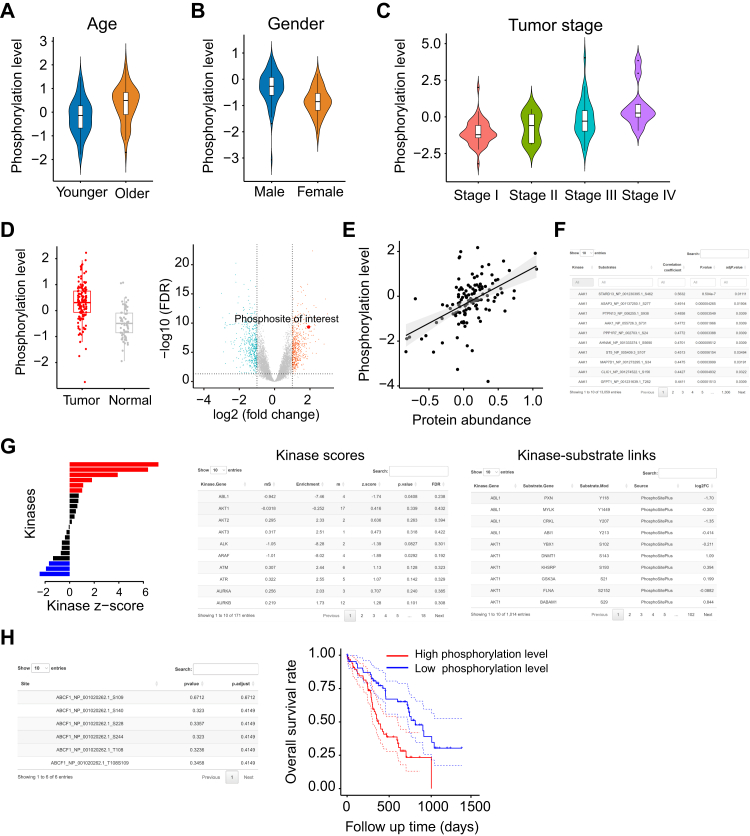
**Phosphoproteome analysis module of iProPhos.***A*–*C*, iProPhos provides clinical feature–related analysis for phosphorylation level, including age (*A*), gender (*B*), and tumor stage (*C*). *D*, iProPhos presents differential phosphorylation level analysis results with *boxplots* or *volcano plots*. *E*, iProPhos provides correlation analysis between protein abundance and the phosphorylation level of the specific phosphosite. *F*, iProPhos conducts correlation analysis between protein abundance of kinases and phosphorylation levels of substrates. *G*, iProPhos estimates the changes in kinase activities in tumor samples compared to normal samples. The *bar pl*ot shows the normalized score for each kinase (z-score), weighted by the number of identified substrates. *Red* means significant activation in tumors, *blue* represents significant inactivation, and *black* denotes kinases with nonsignificant scores; the kinase scores table lists enrichment scores of all kinases; kinase-substrate links table shows the kinase and substrate relationships collected from the selected dataset. *H*, iProPhos performs overall survival analysis based on the phosphorylation level. The table shows the survival analysis results for all detected phosphosites of a given protein; Kaplan–Meier curves show the survival analysis result for a certain phosphosite. The statistical significance is determined by log-rank test.

iProPhos can perform phosphoproteomics-related analyses and integrate with proteomics data, gaining insights into the aberrant phosphorylation events associated with tumorigenesis and progression. The clinical feature-related analysis for phosphorylation level, including age, gender, and tumor stage (Fig. 3, *A*–*C*), and differential analysis comparing phosphorylation level of phosphosites between tumor and normal samples (Fig. 3*D*), are the same as those in the “Proteome Analysis” module. Therefore, these features are not repeated here.

Studying the correlation between protein abundance and the phosphorylation levels of phosphosites provides new insights into the regulatory networks and mechanisms driving tumor progression. iProPhos can evaluate the correlation not only between proteins and their phosphosites but also between proteins and phosphosites on different proteins (Fig. 3*E*).

The abundance of some kinases correlates with their known substrate phosphorylation levels (*e.g.*, CDK1, CDK2) (28, 29). iProPhos can investigate correlations between the protein abundance of kinases and the phosphorylation levels of substrates. The results are presented with the table consisting of kinase-substrate links and the corresponding correlation coefficient and *p* value (Fig. 3*F*). These analyses can provide potential kinase-substrate pairs and reveal the complexity of signaling pathways and networks. The kinase list was collected from PhosphoSitePlus (30) (http://www.phosphosite.org), a well-established resource containing expert-curated annotations, and NetworKIN (31) (https://networkin.info/), which consists of predicted kinase-substrate relationships obtained by integrating various data sources, including sequence-based predictions, experimental kinase-substrate relationships, and PPI networks.

iProPhos can estimate changes in kinase activity based on collective phosphorylation changes of identified substrates using the kinase-substrate enrichment analysis (KSEA) algorithm (32). Differential phosphorylation analysis is performed between tumor and normal samples for a given tumor type using the R package limma, and the results are analyzed using the KSEA module. Users can choose the data sources of the kinase-substrate relationship. Among them, PhosphoSitePlus includes only experimentally verified kinase-substrate relationships, which is recommended for more conservative results. In contrast, NetworKIN provides the predicted relationships. iProPhos presents the results with graphic and tabular formats (Fig. 3*G*). The kinase scores are displayed with bar plots; kinases labeled red are predicted to be significantly activated in tumors, kinases labeled blue are predicted to be significantly inactivated, and black indicates nonsignificance. The list of kinase scores provides the enrichment scores of kinases with at least one identified substrate in the selected dataset, and the list of kinase-substrate links provides the kinase-substrate relationships collected from the selected dataset.

Exploring the correlation between phosphorylation levels and clinical outcomes is valuable for further studies. iProPhos generates a convenient table that provides survival analysis results for all detected phosphosites in a given protein (Fig. 3*H*). Using this function, users can easily select phosphosites that significantly correlate with survival outcomes. iProPhos provides the Kaplan–Meier survival curve plot using the log-rank test (Fig. 3*H*). The grouping methods and colors are customizable.

### iProPhos can Identify Potential Drug Targets

**Fig. 4:**
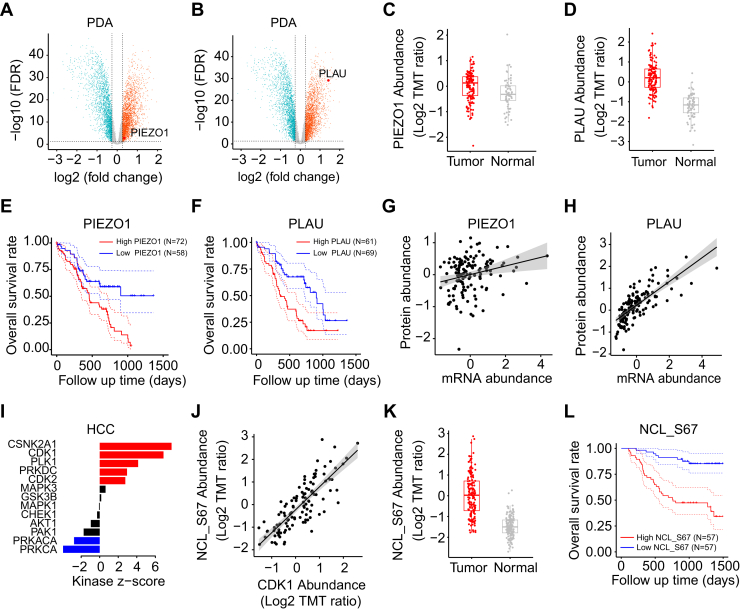
**iProPhos could reveal potential drug targets and infer crucial phosphosites and their upstream kinases.***A* and *B*, *volcano plots* show that PIEZO1 (*A*) and PLAU (*B*) are significantly upregulated in PDA (Benjamini–Hochberg [BH] adjusted *p* < 0.05). *C* and *D*, PIEZO1 (*C*) and PLAU (*D*) show significantly higher expression in PDA. *E* and *F*, survival analysis of PDA patients based on the protein abundance of PIEZO1 (*E*) and PLAU (*F*). *G* and *H*, Pearson’s correlation analysis between mRNA and protein abundance of PIEZO1 (*G*) and PLAU (*H*). *I*, evaluation of kinase activity changes by kinase-substrate enrichment analysis in HCC. *Red* means significant activation in tumors, *blue* represents significant inactivation, and *black* denotes kinases with nonsignificant scores. *J*, Pearson’s correlation analysis between CDK1 abundance and the phosphorylation level of NCL-S67. *K*, *boxplot* shows that phosphorylation level of NCL-S67 is significantly upregulated in HCC. *L*, survival analysis of HCC patients based on the phosphorylation level of NCL-S67. HCC, hepatocellular carcinoma; PDA, pancreatic ductal adenocarcinoma.

Proteomics data provide an excellent opportunity to identify candidate proteins for future clinical treatments, potentially leading to beneficial outcomes. iProPhos has the ability to screen candidate targets. Take the instance of pancreatic ductal adenocarcinoma, which is a highly aggressive malignancy with the low survival rate (33, 34). First, we conducted differential expression analysis between tumor and normal samples using the limma algorithm based on protein abundance. To control the false discovery rate, we used Benjamini–Hochberg (BH) correction for all proteins and the same correction applies to all results in this article. Second, the Food and Drug Administration approved or candidate drug targets were extracted from the Human Protein Atlas. Overlapping proteins between drug targets and differentially expressed proteins (BH adjusted *p* < 0.05) were selected for further analysis. Then, we focused on two proteins, PIEZO1 and PLAU, which were significantly upregulated in tumors (Fig. 4, *A* and *B*). PIEZO1 is a mechanosensitive ion channel, which transduces various mechanical stimulations into electrochemical signals (35), and PLAU is a serine protease, which promotes fibrinolysis and degradation of extracellular matrix (36, 37). In addition, we also employed the Wilcoxon test to validate the differences in protein abundance (PIEZO1, BH adjusted *p* = 1.3e-04; PLAU, BH adjusted *p* = 1.6e-22; Fig. 4, *C* and *D*). Furthermore, we performed survival analysis to investigate the possibility of these two proteins as drug targets. Based on the optimal cut-off point, we classified patients into high- and low-expression groups and found a significant survival difference between them. The high-expression group exhibited a poorer survival outcome than the low-expression group (log-rank test, BH adjusted *p* = 0.027, and BH adjusted *p* = 0.014 for PIEZO1 and PLAU, respectively; Fig. 4, *E* and *F*). Moreover, we found that PLAU genes exhibited significantly positive correlation between mRNA expression and its protein abundance, while PIEZO1 showed a weaker correlation (R = 0.75, BH adjusted *p* = 4.5e-24 for PLAU; R = 0.24, BH adjusted *p* = 7.7e-03 for PIEZO1; Fig. 4, *G* and *H*). These findings highlight the importance of direct exploration of protein expression profiles, as relying solely on mRNA expression may overlook the critical information regarding protein regulation and function.

In summary, iProPhos is a valuable tool that enables researchers to conveniently explore cancer proteomics and identify potential drug targets.

### iProPhos can Identify Crucial Phosphosites and Their Upstream Kinases

The identification of kinase-substrate relationships is crucial for understanding phosphorylation signals. Take the example of hepatocellular carcinoma (HCC), iProPhos inferred kinase activity changes using the KSEA method. Kinase-substrate annotations were obtained from PhosphoSitePlus and NetworKIN, and the NetworkKIN score cut-off was set to 8. Results showed a significant upregulation of the activity of CSNK2A1, CDK1, PLK1, PRKDC, and CDK2 in HCC (Fig. 4*I* and supplemental Table S3). CDK1 plays a critical role in cell cycle progression, and the overactivation of CDK1 has been identified to be closely associated with tumorigenesis (38). NCL-S67 was inferred to be the substrate of CDK1 (supplemental Table S4). We further investigated the correlation between the protein abundance of CDK1 and the phosphorylation level of NCL-S67. The results demonstrated a significantly positive correlation between them (R = 0.81, BH adjusted *p* = 3.3e-25; Fig. 4*J*). We also found that the phosphorylation level of NCL-S67 was significantly upregulated in HCC compared with matched normal samples (Wilcoxon test, BH adjusted *p* = 2.1e-37; Fig. 4*K*). Next, we performed survival analysis using the median phosphorylation level of NCL-S67 as the cut-off value, finding that the high-expression group had a worse prognosis (log-rank test, BH adjusted *p* = 1.8e-03; Fig. 4*L*). These results demonstrate that iProPhos is a powerful tool for discovering critical phosphorylation events, which can ultimately improve our understanding of disease biology.

## Discussion

Mass spectrometry–based proteomics is a widely effective method for identifying, characterizing, and quantifying proteins. Proteins play an indispensable role in biological processes, and PTMs of proteins can regulate the biological functions of cells. The presence of PTMs increases the chemical diversity and complexity of the proteins, making comprehensive analysis of proteomics crucial for understanding cancer biology. However, genomics and transcriptomics methods fail to capture information about PTMs, making proteomics the sole approach for large-scale study of these modifications.

In high-throughput proteomics, analyzing and interpreting quantitative results to propose biological or clinical hypotheses is an ongoing challenge. Currently, there is a scarcity of comprehensive analysis and visualization tools for proteomics data, particularly in phosphoproteomics. Therefore, we have developed iProPhos, a user-friendly web server that provides a comprehensive and integrated analysis platform for proteome and phosphoproteome data. iProPhos can not only separately explore proteomics/phosphoproteomics differences between sample groups, but also integrate proteomics with transcriptomics or phosphoproteomics. iProPhos has the competency of hosting more abundant analyses such as differential expression, patient survival, clinical feature-related, kinase-substrate enrichment, and correlation analyses, including protein–protein, mRNA-protein, and kinase-substrate correlations. In addition, iProPhos provides downstream functional enrichment analysis and PPI network analysis, enabling researchers to gain deeper insights into the functional implications and interactions of the proteins of interest. The extensive analyses provided by iProPhos, accessible with only a few clicks, enable researchers without programming skills to perform multiple analyses. Moreover, iProPhos provides a series of optional parameters for plotting and statistical analysis, helping generate publication-ready figures.

Most existing analysis tools focus on the transcriptome, but protein and mRNA levels exhibit discrepancies in certain genes (8, 39, 40). A previous study explored mRNA-protein correlations across 32 normal human tissues for each gene, finding that the median Spearman correlation was 0.46 (41). It is suggested that proteomics data provide additional information missing in transcriptomic studies, facilitating a more comprehensive understanding of tumor development and the discovery of novel therapeutic strategies. Moreover, phosphoproteomics studies and their integrated analysis with proteomics data further enrich the breadth of the study, enabling the exploration of critical phosphorylation events and kinase-substrate relationships, which fill the gaps in a single-layer analysis and enhance our understanding of the molecular mechanisms underlying the disease.

Due to the limitation of available datasets integrating transcriptome, proteome, and phosphoproteome, iProPhos has collected omics data from only 12 cancer types. In the future, we will continue to incorporate more tumor proteomics datasets, including different cancer types. Moreover, we consider integrating proteomics data from various cancer cell lines in the next version, as these models provide valuable insights into understanding cellular processes and the underlying factors driving carcinogenesis. iProPhos currently provides correlation analysis for paired proteins, but it cannot identify biological networks based on pairwise correlations. In future iterations, iProPhos will provide weighted gene coexpression network analysis to find highly correlated protein clusters and identify hub proteins within those clusters. Furthermore, iProPhos will utilize advanced machine learning algorithms to enhance the efficiency and accuracy of biomarker identification. iProPho will be updated continuously and provide researchers with a powerful tool for studying the role of proteins and PTMs in tumor progression and further leveraging the value of big data in cancer proteomics.

In summary, iProPhos is an intuitive and time-saving tool for the cancer community to dissect oncogenic signaling networks and address unmet clinical needs. Its comprehensive analysis capabilities, integration of different omics data, and potential for biomarker identification make it a valuable asset to unravel the complexities of cancer biology and develop novel therapeutic interventions.

## Data Availability

iProPhos is free and open to all users, with no login requirement. Transcriptome, proteome, phosphoproteome, and clinical data across 12 cancer types were sourced from the CPTAC data portal (https://proteomic.datacommons.cancer.gov/pdc/). The code for producing iProPhos will be deposited to GitHub (https://github.com/longLabzj/iProPhos/), and will be publicly available upon publication.

## Supplemental Data

This article contains supplemental data.

## Conflict of interest

The authors declare no competing interests.
